# Targeting Beta Amyloid: A Clinical Review of Immunotherapeutic Approaches in Alzheimer's Disease

**DOI:** 10.1155/2012/628070

**Published:** 2012-01-15

**Authors:** Kasia Lobello, J. Michael Ryan, Enchi Liu, Gregory Rippon, Ronald Black

**Affiliations:** ^1^Department of Clinical Sciences, Pfizer Inc., Collegeville, PA 19426, USA; ^2^Janssen Alzheimer Immunotherapy Research & Development, LLC., South San Francisco, CA 94080, USA

## Abstract

As the societal and economic burdens of Alzheimer's disease (AD) continue to mount, so does the need for therapies that slow the progression of the illness. Beta amyloid has long been recognized as the pathologic hallmark of AD, and the past decade has seen significant progress in the development of various immunotherapeutic approaches targeting beta amyloid. This paper reviews active and passive approaches aimed at beta amyloid, with a focus on clinical trial data.

## 1. Introduction

Alzheimer's disease (AD) is by far the most common form of dementia, and the social and economic burdens of AD continue to mount. In 2010, an estimated 36 million people worldwide were living with dementia—a number that is projected to increase to 66 million in 2030, and 115 million in 2050 [1]. According to the World Alzheimer Report, the worldwide cost of dementia is estimated at USD $604 billion for 2010 [1], and according to one model, this cost has increased by 34% between 2005 and 2009 [2]. These statistics must be considered in parallel with the immeasurable emotional and psychological burdens that AD places on patients and families.

 Significant progress in the treatment of AD has been made since the initial description of the disease by Alois Alzheimer in 1907 [3]. Cholinesterase inhibitors and memantine are potential therapies for the management of many cognitive symptoms of AD, but these neurotransmitter-based approaches do not address the underlying pathology of the illness, and ultimately fail to prevent its progression. The pathologic triad of AD—the accumulation of toxic beta amyloid with the formation of extracellular beta-amyloid-containing plaques, the development of intracellular neurofibrillary tangles, and the degeneration of cerebral neurons—provides numerous potential targets for disease-modifying therapies. Multiple lines of evidence now suggest, however, that it is the production and/or deposition of toxic forms of beta amyloid, along with the slowing of beta-amyloid clearance, that act as the central and primary events in AD pathogenesis, while neurofibrillary tangle formation and neuronal cell death occur downstream in this amyloid cascade [4–6]. Recent *in vitro* work has demonstrated that beta-amyloid dimers (the major form of soluble oligomers in the human brain) isolated from patients with AD induce both the abnormal phosphorylation of tau that is characteristic of AD and the degeneration of neurites, providing further confirmation of the pivotal role of beta amyloid in the pathogenesis of AD [7]. The search for a disease modifying therapy—one that affects underlying pathology and has a measurable and long-lasting effect on the progression of disability—has thus been aimed primarily at the study of beta amyloid.

 The demonstration of disease modification is best supported by both clinical and biomarker endpoints. A biomarker is an objectively measured characteristic that can be evaluated as an indicator of normal biologic processes, pathogenic processes, or pharmacologic responses to a therapeutic intervention [8]. Several potential biomarkers have been identified in AD and are currently under investigation in interventional clinical trials. These biomarkers should be reflective of changes in the pathology of the AD brain, such as cerebral beta-amyloid deposition, abnormal phosphorylation of tau, or neurodegeneration.

 Recent advances in positron emission tomography (PET) imaging have made possible the *in vivo* detection and quantification of beta amyloid using amyloid-specific ligands, such as the ^11^C Pittsburgh Compound B (PiB) [9, 10]. Elevated levels of tau protein in the cerebrospinal fluid (CSF) are markers of active neuronal degeneration [11], while levels of abnormally phosphorylated tau (P-tau) appear to correlate with the quantity of neurofibrillary tangles in the brain, suggesting that CSF P-tau may serve as an *in vivo* biomarker of the neurofibrillary pathology of AD [12]. Magnetic resonance imaging (MRI)- based measures of cerebral atrophy, most likely the result of excessive neuronal death, correlate closely with the rate of neuropsychological decline in patients with AD [13]. These and other biomarkers will likely play an important role in demonstrating the effect of any therapy on cerebral amyloid and the downstream processes that are affected through beta-amyloid removal.

## 2. Active and Passive Immunotherapeutic Approaches to Beta-Amyloid Clearance

**Figure 1:**
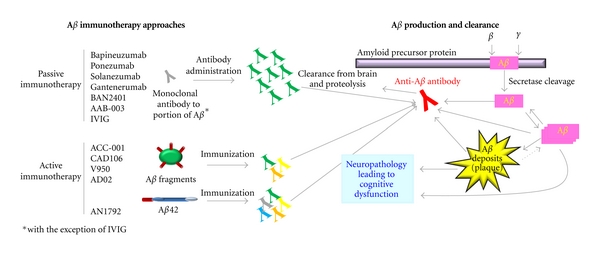
Passive and active immunotherapeutic approaches to beta-amyloid clearance. Beta-amyloid immunotherapeutic compounds currently in clinical trials utilize anti-beta-amyloid antibodies, generated through either passive or active immunotherapy approaches (left), to target beta amyloid and promote its clearance from the brain and proteolysis (right), potentially reversing the neuropathology that leads to cognitive dysfunction. A*β*: beta amyloid.

While numerous strategies have been developed to limit cerebral beta-amyloid deposition and/or facilitate beta-amyloid clearance, the most extensive preclinical and clinical experience to date has come from immunotherapeutic approaches, which can be broadly classified as either active or passive (Figure 1).

 Passive immunotherapy refers to the direct administration of anti-beta-amyloid antibodies, obviating the need for patients to mount an antibody response. Passive immunotherapy in the form of specifically designed monoclonal antibodies allows for the precise targeting of beta-amyloid epitopes. In contrast, active immunotherapy involves the administration of either full-length beta-amyloid peptides or peptide fragments to activate the patient's immune system in order to produce anti-beta-amyloid antibodies. The beta-amyloid peptides or peptide fragments can be conjugated to a carrier protein and may be administered with an adjuvant in order to help stimulate the immune response. As active immunotherapy relies on the patient's own immune response, the extent and nature of anti-beta-amyloid antibody production is likely to vary among individuals, and some patients may not be able to mount a meaningful antibody titer. Active immunotherapy can induce an oligoclonal (as opposed to monoclonal) response with antibodies that differ with respect to their binding affinity for a number of toxic beta-amyloid species. Unlike passive immunotherapy, which has to be readministered at frequent intervals, active immunotherapy has the potential to produce persistent levels of anti-beta-amyloid antibody titers with less-frequent administration.

## 3. Insights from Preclinical Studies

An extensive body of preclinical work (summarized briefly here and reviewed more extensively elsewhere) [14] provides support for an immunotherapeutic approach to beta-amyloid lowering in AD. In 1999, Schenk et al. published a seminal study demonstrating that the administration of beta amyloid_42_ prevented beta-amyloid plaque formation in platelet-derived growth factor promoter (PDAPP) transgenic mice, a mouse model which overexpresses human amyloid precursor protein [15]. Animals treated with this active immunotherapy also demonstrated a marked attenuation in neuritic dystrophy and astrogliosis [15]. Older mice that had already developed some neuropathologic changes at the time of treatment showed a reduction in AD-like neuropathology as compared with older nontreated controls [15]. Schenk's pathology-focused work was followed by the demonstration that beta-amyloid vaccination protected “double transgenic” (APP + PS1) mice from developing the learning and memory deficits that normally occurred in this animal model [16]. Vaccinated double transgenic mice performed as well as non-transgenic controls on the radial-arm water-maze test, suggesting that vaccination may have the potential to restore the wild type phenotype. The ability of beta-amyloid vaccination to attenuate beta-amyloid pathology and behavioral deficits has also been demonstrated in other transgenic models [17].

 In parallel with the active immunotherapeutic approaches described above, preclinical studies utilizing passive immunotherapy spearheaded by Bard et al. established that peripherally administered anti-beta-amyloid antibodies enter the central nervous system and bind to amyloid plaques in PDAPP mice, resulting in a plaque reduction of up to 86% as compared with untreated controls [18]. Plaque clearance was shown to occur through fragment crystallizable (Fc) receptor-mediated phagocytosis by microglial cells, with no evidence of T-cell response activation [18]. Additional work by Wilcock et al. confirmed that administration of anti-beta-amyloid antibodies resulted in the activation of brain microglia (as evidenced by microglial expression of CD45 and the Fc_*Υ*_ receptor), reduced brain beta-amyloid deposits, and improved performance on the Y-maze behavior task in APP transgenic mice [19]. Recent *in vitro *findings demonstrate that antibodies directed at the N-terminal of beta amyloid neutralize the cytoskeletal alterations that are induced by beta-amyloid dimers [7] and that the murine form of bapineuzumab (3D6) interacts with soluble beta-amyloid species. The murine form of bapineuzumab was also effective at neutralizing several *in vitro* and *in vivo* measures of synaptotoxicity in preclinical models [20].

Although Fc receptor-mediated phagocytosis is believed to play a role in immunotherapy-induced beta-amyloid clearance, other studies have demonstrated that Fc receptor interactions are not necessarily required for beta-amyloid removal [21, 22]. These experiments suggest that other mechanisms may also be involved in the antibody-mediated clearance of beta amyloid with active and passive immunotherapy. One of these proposed mechanisms speculates that anti-beta-amyloid antibodies exert their effect not in the brain but rather in the periphery, where they bind to circulating beta-amyloid molecules and reduce the free concentration of beta amyloid in the blood. According to this “peripheral sink” hypothesis, the equilibrium across the blood-brain barrier is then altered to favor the next efflux of beta amyloid from the brain [23]. Another hypothesis proposes that the binding of anti-beta-amyloid antibodies to the beta-amyloid molecule alters its conformation so that it is less likely to form the fibrillar aggregates associated with AD pathology [24].

Preclinical data is also available for human intravenous immunoglobulin (IVIG). Magga et al. demonstrated that peripherally administered IVIG penetrated the blood-brain barrier and bound to beta-amyloid deposits in mouse brain [25]. In addition, IVIG obtained from the plasma of healthy human volunteers protected mouse hippocampal neurons from beta-amyloid toxicity *in vitro* [25]. The effects of IVIG may be due in large part to the presence of naturally occurring anti-beta-amyloid antibodies, which are abundant in human plasma but decline with age and advancing AD [26]. Numerous other mechanisms of action have been proposed for IVIG, including complement binding, interference with B-cell differentiation, and cytokine modulation [27].

## 4. Clinical Trials with Active Immunotherapy

### 4.1. AN1792

#### 4.1.1. Phase 1 Trial

Following on the promising preclinical results described above, AN1792, a synthetic beta-amyloid peptide, was the first active amyloid immunotherapy tested in clinical trials [28]. The initial study randomized 80 subjects with mild-to-moderate AD; 64 subjects received AN1792 with QS-21 (adjuvant) and 16 received QS-21 alone [28]. Injections were administered 4 times over a 24-week period, with an optional extension phase that allowed subjects to receive up to 4 additional injections over a total follow-up time of 84 weeks [28].

 Of the 64 subjects who received AN1792, 53% developed a positive anti-AN1792 antibody response (defined as an antibody titer ≥1 : 1,000) at one or more points during the trial [28]. Exploratory efficacy analyses showed no difference between rates of cognitive decline in treated and control groups as measured by the Alzheimer's Disease Assessment Scale-Cognitive (ADAS-cog) and Mini-Mental State Examination (MMSE) [28]. At week 84, however, patients who had received AN1792 showed less functional decline (as measured by the Disability Assessment for Dementia [DAD]) than those treated with QS-21 alone (adjusted mean values −14.15 versus −36.42, *P* < 0.002) [28].

Although the vast majority of adverse events (AEs) reported during the initial study were either mild or moderate in nature, one patient treated with AN1792 developed severe dizziness, disorientation, and functional deterioration [28]. This patient died approximately 1 year after her fifth and final injection of AN1792, and a few weeks after dose administration in the phase 2a trial was halted due to cases of meningoencephalitis (see below) [28]. The patient's postmortem examination revealed changes consistent with T-lymphocytic meningoencephalitis and was also significant for extensive cortical areas devoid of beta-amyloid plaques [29].

#### 4.1.2. Phase 2a Trial and Meningoencephalitis

The phase 2a study of AN1792 in mild-to-moderate AD randomized 300 patients to receive AN1792 (QS-21) and 72 patients to receive placebo [30]. Dosing was halted after 18 AN1792 (QS-21)-treated patients (6% of all patients who received active therapy) developed aseptic meningoencephalitis [30]. AN1792-associated meningoencephalitis was variable with respect to clinical presentation, severity, and resolution. Most patients developed progressive confusion, lethargy, and headache [31]. Other reported signs and symptoms included fever, nausea, vomiting, seizures, and focal neurologic signs [31]. Meningoencephalitis developed from 5 to 168 days after the last injection of AN1792, with a median latency of 40 days [31]. While most patients experienced a monophasic illness, 4 patients developed a relapse following the initial resolution of meningoencephalitis, and 2 of these relapses were severe [31]. Recovery was reported in 12 of the 18 patients, while 6 patients were noted to have persistent sequelae at the conclusion of the trial [31]. No additional cases of meningoencephalitis were reported over a 4.6-year follow-up study of subjects previously enrolled in the 2a trial [32]. No cases of meningoencephalitis occurred in the placebo-treated patients [31, 32].

Geometric mean serum anti-AN1792 antibody titers were not significantly higher in those patients who developed meningoencephalitis than in those who did not [30]. Five of the 18 patients with meningoencephalitis did not meet the antibody responder criterion (defined here as a serum anti-AN1792 IgG [total] titer ≥1 : 2,200 at any time after injection), and one patient with meningoencephalitis had serum IgG titers <1 : 50 over the entire study course [30]. No correlation was established between antibody titers and time-to-symptom onset, severity of illness, or relapse [31].

 MRI findings in patients with meningoencephalitis were also variable, ranging from subtle meningeal enhancement to cerebral edema and extensive parenchymal signal abnormalities with a predominantly posterior distribution [31]. No hemorrhagic findings (such as microhemorrhages or larger hemosiderin deposits) were reported [31]. CSF analysis was performed in 17 of the 18 cases; 16 of these revealed a lymphocytic pleocytosis, with WBC counts ranging from 15 to 130 cells/mL, and CSF protein from 0.33 to 3.1 g/L (33–310 mg/dL) [31]. Glucose levels were within normal limits [31]. IgG levels were elevated in 3 of 4 patients tested, and oligoclonal bands were reported in 2 of the 18 cases [31].

 One possible factor in the development of meningoencephalitis (which had not occurred in preclinical studies) in this subset of AN1792 (QS-21)-treated patients stems from the introduction of polysorbate 80 into the formulation [30]. At the late stages of the phase 1 trial described above, polysorbate 80 was added to prevent AN1792 (QS-21) precipitation [29]. The single subject who developed meningoencephalitis in the phase 1 study did so approximately 36 days after receiving the fifth dose of AN1792 (QS-21), which was also the first dose using the altered formulation [29]. It is possible that the addition of polysorbate 80 resulted in an increased exposure of amyloid-beta_1−42_ amino acids to epitopes capable of mounting an inflammatory T-cell response [30, 33]. Peripheral blood mononuclear cell isolates from patients who received AN1792 were analyzed for cytokine response to beta-amyloid-derived peptides using enzyme-linked immunosorbent spot (ELIspot) assays. These assays showed a difference in the quality of the T-cell response induced by the two different formulations of AN1792, with isolates from patients who received the polysorbate 80-containing formulation more likely to exhibit a beta-amyloid-specific proinflammatory Th1 response. The specificity of the antibody response did not differ between the two formulations and was directed almost exclusively to the N-terminus [33]. Neuropathologic examination of one case of meningoencephalitis revealed a perivascular T-cell infiltrate with a lack of B lymphocytes, as well as microglial activation and multinucleated giant cells [34]. Lymphocyte distribution was most prominent in the temporal cortex, hippocampus, and amygdale and did not match the observed distribution of beta-amyloid clearance. Lymphocyte distribution did appear to correspond with the finding of collapsed plaques, which were characterized by abnormal morphology and composed of dense amyloid cores surrounded by activated microglial cells. This colocalization of lymphocytic infiltrates and abnormal plaques may suggest that meningoencephalitis was related to abnormal beta-amyloid processing and not to beta-amyloid clearance [34].

#### 4.1.3. Immunologic and Clinical Outcomes

At the time of discontinuation of dosing due to meningoencephalitis, over 90% of patients in the phase 2a trial had received 2 of the planned 6 injections of AN1792 (QS-21)/placebo [30]. The study was amended to allow safety follow-up for at least 9 months after the last injection [30]. Despite the limited number of administered injections, approximately 20% of the subjects treated with AN1792 (QS-21) were classified as antibody responders [30].

 As expected, the AN1792 phase 2a study demonstrated no differences between treatment groups in the majority of the cognitive, functional, and global change scores, which became exploratory measures when dosing was discontinued [30]. However, the composite Neuropsychological Test Battery (NTB) z-score, as well as other NTB component scores including the memory scores, showed less worsening in the antibody responder group compared with the placebo group (*P* = 0.020) at month 12 [30]. Moreover, there was a direct relationship between mean antibody titers and the overall composite NTB z-score, as well as NTB z-scores for all memory, immediate memory, and delayed memory, indicating greater improvements from baseline in patients with higher antibody titers [30].

The short-term results of the abbreviated phase 2a trial must be viewed in the context of the long-term follow-up study, which enrolled 159 of the patients who had originally participated in the AN1792 phase 2a trial, including 25 antibody responders, 104 low/nonresponders, and 30 placebo-treated patients [32]. Of the 19 antibody responders who submitted a blood sample for testing, 17 (89.5%) demonstrated a persistently positive anti-AN1792 antibody titer approximately 4.6 years after their last injection of AN1792 [32]. Although these titers were low (geometric mean of 1 : 331.5), they seemed sufficient to provide meaningful long-term benefits in some of the efficacy findings that were examined in this population [32]. As compared with placebo-treated patients, antibody responders had a 25% lower decline in activities of daily living as assessed by the DAD, a reduction in decline on the Rey Auditory Verbal Learning component of the NTB, and 20% less decline on the Clinical Dementia Rating Scale Sum of Boxes (CDR-SOB) [32]. The differences between placebo-treated patients and antibody responders were statistically significant for the first 2 of these assessments (*P* = 0.015 and *P* = 0.046, resp.) [32]. After approximately 4.6 years of follow-up, 76% of antibody responders were living in their own home and 16% were living in long-term care institutions; the percentages for placebo-treated patients were 53% and 30%, respectively [32]. On the Dependence Scale, antibody responders had a 17.6% lower mean score in caregiver dependence compared with placebo-treated patients (*P* = 0.033). No significant differences between antibody responders and placebo-treated patients were noted on the composite NTB z-score, the MMSE, or the ADAS-Cog [32].

 It is important to note that while the dependence measures (such as DAD and institutional status) could be assessed based on caregiver input, cognitive measures had to be obtained from patients directly [32]. Many patients in the long-term follow-up study were unable or unwilling to provide responses to these cognitive assessments, resulting in a proportionally higher percentage of missing data for these endpoints, as compared with the functional scales [32]. The extent of missing functional data may have contributed to the lack of clear placebo/treatment differences on cognitive endpoints [32].

#### 4.1.4. Biomarkers

Along with the potential clinical efficacy signals described above, several important biomarker changes were observed in the AN1792 phase 2a trial. Ten antibody responders and 11 placebo-treated patients underwent pre- and postbaseline CSF analysis [30]. A statistically significant reduction in CSF tau was seen in the antibody responder group, but there was no treatment effect on CSF beta-amyloid levels [30].

 Baseline and post-treatment brain MRI scans (obtained either at month 12 or at early termination) were available for 288 of the 372 patients who participated in the study, and were used to determine whole-brain, ventricular, and hippocampal volumes [35]. The change in whole-brain boundary shift interval (BSI) over the treatment period was greater in the antibody responders than in the placebo group (*P* = 0.007), indicating a greater loss of brain volume in the antibody responder group [35]. Antibody responders also had a significantly greater increase in ventricular volume than placebo-treated patients (*P* < 0.001) [35]. Anti-AN1792 IgG serum titers correlated with the percent change in whole-brain BSI (Pearson correlation coefficient *r* = 0.293; *P* = 0.003) and ventricular BSI (*r* = 0.472; *P* < 0.0001) in treated patients who had titers ≥1 : 100 [35].

 Both placebo-treated patients and antibody responders exhibited a decrease in hippocampal volumes over the study period, with no significant difference between the 2 groups [35]. Whole-brain, ventricular, and hippocampal volumes in nonresponders did not differ from those of placebo-treated patients [35]. The AN1792 follow-up study [32] showed no significant difference in whole-brain or hippocampal volume changes from baseline between antibody responders and placebo-treated patients at a mean follow-up of 4.6 years, but the number of patients for whom MRI data was available was small. The antibody responders, however, showed a greater increase in ventricular volume than that seen in placebo-treated patients (*P* = 0.021) [32].

 The etiology and clinical significance of the above-described changes in MRI-measured brain and ventricular volumes remains unclear. The suggestion that AN1792 caused an accelerated rate of neurodegeneration in antibody responders is unsupported, given the lack of worsened clinical decline (and in light of the potential signals of clinical benefit) in patients who developed positive titers [35]. An alternative explanation is that at least some of the cerebral volume loss can be accounted for by the removal of beta-amyloid plaques in antibody responders and/or by parenchymal/CSF fluid shifts that may have occurred in parallel with shifts in beta amyloid [35]. The latter two hypotheses are now supported by multiple autopsy cases that have been performed on AN1792 responders, which clearly demonstrate effective beta-amyloid clearance (see below).

#### 4.1.5. Pathologic Findings

In 2006, Nicoll et al. published the neuropathologic findings of 3 patients who had received between 2 and 5 doses of AN1792 (QS-21) [36]. Two of the 3 cases were known to have developed anti-beta-amyloid antibodies over the course of the study, and both of these patients also developed meningoencephalitis [36]. The causes of death in the autopsied cases, however, were nonneurologic in nature (pulmonary embolism, bronchoaspiration, and abdominal aortic aneurysm) [36]. Nicoll et al. compared the findings in the AN1792 (QS-21)-treated cases to 7 untreated cases that met neuropathologic criteria for AD [36].

A marked reduction in beta-amyloid plaque deposition was noted in the temporal cortex of the 2 AN1792 (QS-21) cases that had developed anti-beta-amyloid antibodies: 69% and 89% of the temporal cortex was classified as plaque-free, as compared with <1% in control cases [36]. Plaque removal appeared patchy, with a relatively higher plaque density in the frontal lobes [36]. The antibody nonresponder case demonstrated no plaque-free areas in the temporal and medial frontal cortex but showed some patchy areas of plaque removal elsewhere [36]. Morphologic studies revealed further evidence of plaque clearance in all 3 immunized cases and demonstrated the presence of beta-amyloid granules within lysosomes and activated microglia [36]. Beta amyloid with an intact N-terminus had been cleared effectively, while beta-amyloid species truncated at the N-terminus persisted [36].

 Although both antibody-positive subjects in this series also had a history of meningoencephalitis [36], other neuropathologic examinations have demonstrated plaque clearance in subjects without meningoencephalitis [37]. In contrast to cortical amyloid, vascular amyloid was not removed by active immunization, as all 3 autopsy cases had severe cerebral amyloid angiopathy (CAA) at autopsy [36]. This finding has been confirmed by Patton et al., who examined 2 AN1792-immunized patients and noted that while both compact core and diffuse amyloid deposits were diminished, vascular deposits were relatively preserved or even increased [38].

 Importantly, the work of Nicoll et al. showed that plaque-free cortical regions also exhibited a decrease in the density of dystrophic neurons, although there was no clear evidence of an impact on neuronal tau or neurophil threads [36]. The downstream effects of beta-amyloid immunization were further elucidated by Serrano-Pozo et al., who performed detailed quantitative analyses of hippocampal sections from AN1792-treated patients and compared these with samples from nondemented controls and untreated patients with AD [39]. In addition to the expected clearance of beta-amyloid plaques in immunized patients, Serrano-Pozo et al. demonstrated the normalization of neurite morphology and a significant reduction in the hyperphosphorylation of tau [39]. The ability of AN1792 to reduce tau hyperphosphorylation has also been reported elsewhere [34].

 While the neuropathologic findings discussed above provide compelling evidence for the beneficial effects of active beta-amyloid therapy on both beta-amyloid plaque burden and the downstream effects of beta-amyloid pathology, the clinical significance of beta-amyloid clearance has been challenged by other autopsy studies. Holmes et al. published long-term findings of AN1792-treated subjects up to 5 years after the last injection of AN1792 [40]. When compared with placebo-treated subjects who had also consented to long-term follow-up, immunization had no effect on long-term survival or clinical outcomes, although the number of subjects was small [40]. Eight AN1792-treated patients with AD consented to autopsy, which demonstrated a long-term reduction in mean beta-amyloid load as compared with untreated controls [40]. Seven of these 8 patients, 2 of whom had nearly complete beta-amyloid removal at autopsy, also had severe end-stage dementia at the time of death, leading the authors to conclude that progressive neurodegeneration had occurred despite effective clearance of beta amyloid [40]. It is important to point out, however, that the neuropathologic examinations performed in this sample included only those patients who died, and the findings are therefore not generalizable to those treated patients who survived. As such, the results of this study are in direct conflict with those reported by Vellas et al., who demonstrated a long-term clinical benefit with AN1792 [32].

### 4.2. Active Immunotherapies Currently in Clinical Trials

While studies with AN1792 were discontinued due to the occurrence of meningoencephalitis, the trials paved the way for the many active immunotherapeutic clinical trials currently in progress (Table 1 and Figure 1). In a study of serum samples from patients immunized with AN1792, Lee et al. established that the predominant antibody response in these patients was against the free N-terminus of beta amyloid; specifically, against beta amyloid_1-8_ [41]. Vanutide cridificar (ACC-001) is a conjugate of multiple copies of beta-amyloid_1-7_ peptide linked to a nontoxic variant of diphtheria toxin [42]. Preclinical data indicate that vanutide cridificar generates N-terminal anti-beta-amyloid antibodies without inducing a beta-amyloid-directed T-cell response, and that it reverses cognitive impairment in murine models of AD [42]. Vanutide cridificar is currently in phase 2 clinical trials in mild-to-moderate AD and early AD (NCT01284387; NCT01227564; NCT00479557; NCT00955409; NCT00498602; NCT00752232; NCT01238991; NCT00960531; NCT00959192). Other active immunotherapies currently under study include CAD106 (Novartis, Inc.), V950 (Merck & Co.), and AD02 (AFFiRiS AG/GlaxoSmithKline plc).

#### 4.2.1. CAD106

CAD106 is composed of the beta-amyloid_1-6_ peptide coupled with a Q*β* carrier [43, 44]. A 52-week study with CAD106 included 58 patients with mild-to-moderate AD in 2 cohorts: 50 *μ*g CAD106 or placebo administered at weeks 0, 6, and 18 (cohort 1); or 150 *μ*g CAD106 or placebo at weeks 0, 2, and 6 (cohort 2) [43, 44]. Injection-site erythema was the most frequent AE observed with CAD106 (4% in cohort I; 64% in cohort II); most AEs were mild, and serious AEs were considered unrelated to study medication [43, 44]. CAD106 was associated with an antibody response in 16/24 treated patients in cohort 1 and 18/22 patients in cohort 2 [43, 44]. In 2 52-week, phase 2a studies in 58 patients with mild AD, 150 *μ*g CAD106 was administered subcutaneously at weeks 0, 6, and 12 (study 1), or either subcutaneously or intramuscularly at weeks 0, 2, and 6 (study 2) [45]. Results of study 1 showed antibody response in 20/22 patients. Because the results indicated that week 2 injection did not enhance antibody response, a 0/6/12 week regimen was selected for further study [45]. Two phase 2 studies currently in progress are investigating repeated administration of CAD106 intramuscularly (NCT01097096) or subcutaneously (NCT00956410; NCT01023685).

#### 4.2.2. V950

V950 is a multivalent beta-amyloid vaccine [46]. To date, no clinical data have been presented. Preclinical studies have shown that administration of V950 results in the production of anti-beta-amyloid antibodies in the serum and CSF that recognize pyroglutamate-modified and other N-terminally truncated beta-amyloid fragments [46]. A phase 1 study of V950 in patients with AD is currently underway (NCT00464334).

#### 4.2.3. AFFITOPE AD02

AFFITOPE AD02 is composed of a 6-amino acid peptide that mimics part of the N-terminus of beta amyloid [47]. It is hypothesized that AD02 and other active immunotherapeutic approaches using this technology may have a favorable safety profile because they are nonself and thus do not need to overcome tolerance—their small size prevents autoreactive T-cell activation, and their controlled specificity prevents cross-reactivity with amyloid precursor protein [48]. Phase 1 data showed a favorable safety profile with AD02 and AD01, another AFFITOPE compound [48]. A randomized, multicenter, phase 2 trial with AD02 in patients with early AD is currently recruiting participants (NCT01117818).

## 5. Clinical Trials with Passive Immunotherapy

Passive immunotherapeutic approaches to AD are being investigated in parallel with the active therapies described above. To date, the largest quantity of published data on passive immunotherapy pertains to bapineuzumab, which is being codeveloped by Pfizer Inc. and Janssen Alzheimer Immunotherapy Research & Development, LLC.

### 5.1. Bapineuzumab

#### 5.1.1. Phase 1 Trial

Bapineuzumab is a humanized monoclonal antibody that targets the N-terminal region of beta amyloid [49] (Figure 1). Bapineuzumab at doses of 0.5, 1.5, or 5 mg/kg was first tested in patients with mild-to-moderate AD in a 12-month, single ascending-dose study [49]. The majority of treatment-emergent AEs were mild to moderate in severity and were not considered related to treatment by study investigators [49]. The phase 1 study included protocol-specified periodic MRI monitoring, and MRI abnormalities consistent with vasogenic edema were reported in 3 of the 10 patients randomized to bapineuzumab 5.0 mg/kg [49]. Recently published recommendations from the Alzheimer's Association Research Roundtable Workgroup include the use of the term amyloid-related imaging abnormalities (ARIA) in reference to the spectrum of imaging findings associated with amyloid-lowering therapies and ARIA-edema/effusions (ARIA-E) to refer to findings previously referred to as “vasogenic edema” [50]. The authors have chosen to adopt this terminology for the purposes of this paper. The MRI findings in the bapineuzumab study consisted of hyperintensities on fluid-attenuated inversion recovery (FLAIR) sequences [49]. In one of the 3 cases, the FLAIR abnormality was accompanied by the development of a new microhemorrhage [49]. The MRI abnormalities, with the exception of the microhemorrhage, resolved in all 3 cases over a period of weeks to months [49]. Two of the 3 patients with ARIA-E were asymptomatic, and one patient experienced mild and transient confusion [49]. Two of the 3 patients with ARIA-E underwent CSF analysis. In contrast to the meningoencephalitis cases reported with AN1792, CSF was acellular in both cases, with minor elevations in CSF protein (58.5 and 59.8 mg/dL) [49].

 Plasma levels of bapineuzumab increased over 1-2 hours following an infusion, and plasma half-lives ranged from 21 to 26 days [49]. MMSE was performed as an exploratory efficacy measure, and mean MMSE increased from baseline over the course of the trial at the 0.5 and 1.5 mg/kg bapineuzumab doses (except for month 6 at the 1.5 mg/kg dose) [49]. Mean MMSE decreased in patients on placebo except at month 6 and also decreased in patients who received 5.0 mg/kg of bapineuzumab [49]. At the primary time point (week 16), at the 1.5 mg/kg dose, the treatment versus placebo difference in MMSE (2.6) was statistically significant (*P* = 0.047) in favor of bapineuzumab [49].

#### 5.1.2. Phase 2 Trial

The phase 1 study was followed by a multiple ascending dose trial in which 124 patients with mild-to-moderate AD were randomized to 1 of 4 doses (0.15, 0.5, 1, or 2 mg/kg) of bapineuzumab, and 110 patients received placebo [51]. Study assessments consisted of numerous clinical evaluations (including the ADAS-Cog, NTB, and DAD), safety, tolerability, and biomarkers, including CSF and brain volume [51]. Bapineuzumab or placebo infusions were given every 13 weeks for up to 78 weeks [51]. In the prespecified efficacy analyses (within-dose-cohort differences between bapineuzumab and placebo from baseline to week 78), no significant difference was seen in any of the cohorts on either of the pre-specified primary outcomes (ADAS-Cog or DAD). Exploratory analyses on the overall treatment groups (pooled bapineuzumab versus placebo) revealed trends on the ADAS-Cog and NTB, but not on the DAD or other outcomes [51]. Treatment differences in ADAS-Cog, NTB, and DAD became more apparent when analyses were carried out on the “completer” population [51].

 Post-hoc exploratory efficacy analyses were also carried out by apolipoprotein E *ε*4 (ApoE4) carrier status, following the observation that ARIA-E was more common in ApoE4 carriers (see below) [51]. In the 79 ApoE4 noncarriers, bapineuzumab/placebo treatment differences were observed in several outcomes, including the ADAS-Cog and the NTB, although there was no difference on the DAD [51]. No treatment differences were observed on any of the endpoints in the 146 ApoE4 carriers although potential efficacy signals became apparent in analyses limited to those ApoE4-positive patients who completed the trial [51]. In general, treatment differences began to emerge at approximately month 9 of the trial [51].

 The bapineuzumab phase 2 trial included CSF biomarkers and MRI volumetric endpoints. CSF samples were obtained in 35 study subjects. There were no observed treatment differences in either CSF beta amyloid or total tau levels, but there was a trend towards greater reduction in P-tau in bapineuzumab-treated patients when compared with placebo (*δ* = −9.1 pg/mL; 95% CI, 18.5–0.3; *P* = 0.056) [51]. In a subsequently conducted exploratory pooled analysis including patients from the PET PiB study (described below), when comparing the change from baseline to end-of-study CSF P-tau values, a significant treatment reduction was observed in the bapineuzumab-treated patients compared with patients who received placebo (−7.26 pg/mL, *P* = 0.0270) [52].

 No overall differences between combined bapineuzumab and placebo-treated patients were observed with respect to whole-brain and ventricular volumes as measured by MRI volumetric analyses over 18 months [51]. However, ApoE4 noncarriers treated with bapineuzumab showed less brain volume loss than those on placebo (*δ* = −10.7 mL; 95% CI, 3.4–18.0; *P* = 0.004) [51]. No differences in brain volume were noted in ApoE4 carriers, but the bapineuzumab group had a greater increase in ventricular enlargement than the placebo-treated subjects (*δ* = 2.6 mL; 95% CI, 0.2–5.0; *P* = 0.037) [51].

 The most common AEs (reported in >5% of bapineuzumab patients and occurring at a rate of at least twice that of placebo) included ARIA-E, back pain, anxiety, and paranoia. Other AEs which also occurred more frequently in the bapineuzumab group included deep vein thrombosis, syncope, seizures, vomiting, hypertension, weight loss, skin laceration, gait disturbance, muscle spasm, and pulmonary embolism [51].

 ARIA-E, which was noted in the phase 1 trial, was detected in 12 of the 124 bapineuzumab-treated subjects (9.7%) and none of the placebo-treated subjects in the phase 2 study [51]. As was true in the earlier study, ARIA-E was more likely to occur at higher bapineuzumab doses, with rates of 3.2%, 0%, 10%, and 26.7% for the 0.15 mg/kg, 0.5 mg/kg, 1 mg/kg, and 2 mg/kg doses, respectively [51]. Eleven of the 12 cases of ARIA-E were detected following either the first or second bapineuzumab infusion [51]. Six of the patients with ARIA-E had no clinical symptoms, while 6 patients experienced symptoms such as headache, confusion, vomiting, and gait disturbance [51]. These symptoms were transient although one patient required treatment with steroids [51]. The MRI findings in these ARIA-E cases were consistent with those described in the earlier trial and resolved over a period of several months [51]. The clinical and MRI characteristics of bapineuzumab-associated ARIA-E, along with the lack of evidence of inflammation as illustrated by the CSF findings described above, differentiate bapineuzumab-related ARIA-E from the severe cases of meningoencephalitis that occurred in association with AN1792.

 One of the most unexpected findings in the bapineuzumab phase 2 study was the increased rate of ARIA-E in ApoE4 carriers. Ten of the 12 ARIA-E cases occurred in ApoE4 carriers, and the ARIA-E rates in ApoE4 carriers and noncarriers were 13.5% and 4.3%, respectively [51]. Moreover, among ApoE4 carriers, the rate of ARIA-E increased with the gene dose, with rates of 7.1% in ApoE4 heterozygotes and 33.3% in ApoE4 homozygotes [51]. These findings are particularly intriguing in light of the potential ApoE4-dependent efficacy differences discussed above, and may be due at least in part to the increased load of beta amyloid in ApoE4 carriers, including a higher vascular beta-amyloid burden [51, 53]. It should be noted that the prevalence of the ApoE4 allele appears to vary by geographic location. An estimated 37–43% of Asian and southern European/Mediterranean AD patients are ApoE4 carriers, compared with 58% of patients in North America and 64% in northern Europe [54].

Although its mechanism is unknown, ARIA-E may result from transient increases in cerebral vascular permeability secondary to vascular amyloid clearance [51]. This theory is supported by reports of spontaneously occurring amyloid-related imaging abnormalities similar to those seen in the bapineuzumab trials [55, 56].

#### 5.1.3. Positron Emission Tomography (PET) Carbon-11-Labelled Pittsburgh Compound B (^11^C-PiB) Study

The ability of bapineuzumab to clear cerebral beta amyloid was demonstrated *in vivo *in a trial of patients with mild-to-moderate AD who underwent serial PET scans with carbon-11-labelled Pittsburgh compound B (^11^C-PiB) [57]. PiB is known to bind to aggregated fibrillar beta-amyloid deposits and is therefore a marker of fibrillar beta-amyloid load [9, 58]. PiB also binds to cerebrovascular amyloid [59]. In the trial, 20 patients were randomized to bapineuzumab at 1 of 3 doses (0.5, 1.0, or 2.0 mg/kg) for a total of up to 6 infusions, 13 weeks apart; 8 patients received placebo [57]. The primary outcome measure for the trial was the difference between the pooled bapineuzumab groups and the placebo group in the mean change (from screening to week 78) in the ^11^C-PiB cortical to cerebellar retention ratio [57]. The cerebellum was used as a reference region because it exhibits a relatively low beta-amyloid load in AD [57].

**Figure 2:**
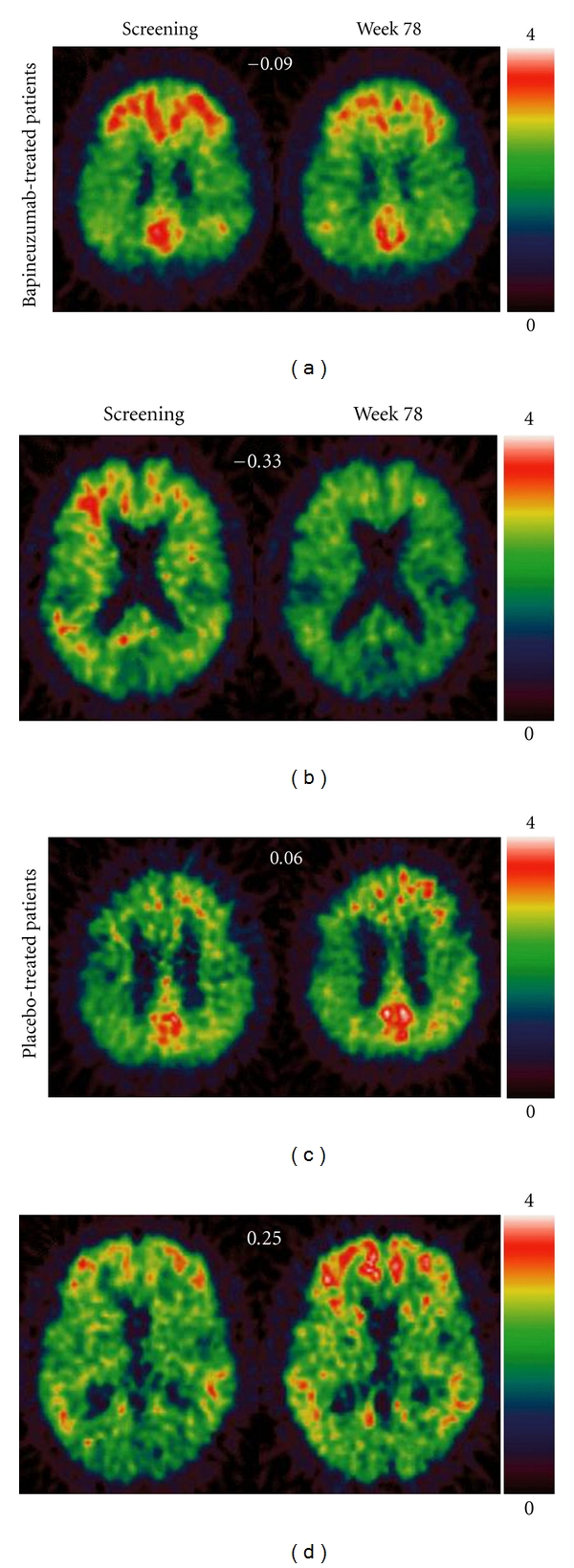
Positron emission tomography (PET) carbon-11-labelled Pittsburgh compound B (^11^C-PiB) images from patients treated with bapineuzumab and those given placebo [57]. *Reprinted from *[57]* with permission from Elsevier.* Changes from screening to week 78 in patients treated with bapineuzumab (a, b) and in patients treated with placebo (c, d). Mean ^11^C-PiB PET changes are shown at the top center of each panel for each patient. The scale bar shows the PiB uptake ratios relative to cerebellum by color. The scans before and after treatment are from MRI coregistered images in the same plane.

 By week 78, the estimated mean ^11^C-PiB retention ratio decreased by 0.09 in the bapineuzumab group and increased by 0.15 in the placebo group, with an estimated treatment difference of −0.24 (95% CI,0.39 to −0.09; *P* = 0.003). This finding correlates to an approximately 25% reduction in cortical beta amyloid in bapineuzumab-treated patients [57] (Figure 2). The extent of beta-amyloid reduction was not clearly dose dependent [57]. Bapineuzumab/placebo differences in ^11^C-PiB retention were statistically significant in all prespecified cortical regions (anterior and posterior cingulate, frontal, temporal, parietal, and occipital cortex) [57]. After adjustment for imbalances in baseline clinical and ^11^C-PiB-binding characteristics, there were no treatment differences noted on clinical or biomarker endpoints between the bapineuzumab and placebo groups [57].

### 5.2. Other Passive Immunotherapies Currently in Clinical Trials

Building on the data presented above, a phase 3 program for bapineuzumab is currently in progress, comprised of 4 trials: 2 studies in ApoE4 carriers (NCT00575055; NCT00676143) and 2 trials in ApoE4 noncarriers (NCT00574132; NCT00667810). There is considerably less published data available on other passive immunotherapeutic approaches, which include solanezumab (Eli Lilly and Company), ponezumab (Pfizer Inc.), gantenerumab (Hoffmann-La Roche, Ltd.), BAN2401 (Eisai Co., Ltd.), and intravenous immunoglobulin (Baxter International Inc. and Octapharma AG). These compounds are described briefly below and in Table 2 (see also Figure 1).

#### 5.2.1. Solanezumab

Solanezumab (LY2062430) is a humanized monoclonal antibody against the mid-domain of beta amyloid [60]. In a study to assess the safety and tolerability of single-dose solanezumab (0.5, 1.5, 4.0, or 10.0 mg/kg) in patients with mild-to-moderate AD, solanezumab was associated with infusion reactions in 2 out of 4 patients receiving the highest dose (10.0 mg/kg) [60]. Serious AEs were not considered to be related to the study medication [60]. There was no evidence of inflammation based on MRI or CSF white blood cells counts, and analysis of C-reactive protein (CRP) in blood samples showed only isolated elevations [60]. In a multicenter, multiple-dose, open-label study in Japan, 33 patients with mild-to-moderate AD received a 400 mg dose of solanezumab intravenously every week, every 4 weeks, or every 8 weeks [61]. Most AEs were mild to moderate; one severe event was reported but was considered unrelated to the study medication [61]. There were no reports of infusion reactions or meningoencephalitis [61]. Studies examining biochemical biomarkers found that plasma and CSF-amyloid_1−40_ and beta-amyloid_1−42_, plasma pyro-Glu 3–42 beta-amyloid (N3pGluA*β*), and plasma and CSF N-terminally truncated beta-amyloid peptide (fragment 2), but not CSF total tau and phosphorylated tau (P-tau_181_), exhibited significant changes in patients receiving solanezumab, indicating the utility of these biomarkers for evaluating the pharmacodynamic effects of solanezumab [60, 62–66]. In addition, there was a correlation between plasma beta-amyloid_1−42_ and assessment of amyloid burden using single photon emission tomography with IMPY [64]. Three phase 3 studies—2 evaluating the effects of solanezumab on disease progression, and one extension study monitoring safety for participants in those studies—are in progress (NCT00904683; NCT00905372; NCT01127633). In addition, a phase 2 study evaluating biomarkers with solanezumab in individuals with or without AD is currently in the recruitment phase (NCT01148498).

#### 5.2.2. Ponezumab

Ponezumab (PF-04360365) is a humanized IgG2deltaA monoclonal antibody that binds to amino acids 33–40 of the beta-amyloid_1−40_ peptide [67]. Two 1-year, phase 1 studies were performed to assess the safety, pharmacokinetics, and pharmacodynamics of ponezumab in patients with mild-to-moderate AD [67–69]. In the first study, which was randomized and double-blinded, patients received placebo or ponezumab 0.1, 0.3, 1, 3, or 10 mg/kg via 2-hour infusion; in the second study, an open-label, parallel-group study, patients received ponezumab 1, 3, 5, or 10 mg/kg via 10-minute infusion [69]. All AEs were mild or moderate, with no serious AEs considered to be related to study drug [67, 68]. In the 2-hour infusion study, one patient receiving ponezumab 10 mg/kg had a mild hypersensitivity reaction, and a preexisting brain lesion showed a slight increase in size in a patient receiving ponezumab 0.1 mg/kg [68]. No new microhemorrhage, ARIA-E, or encephalitis was found in either study [67, 68]. Ponezumab showed linear pharmacokinetics in both studies [67–70]. Low ponezumab concentrations were detected in CSF in 2 out of 8 patients receiving the highest dose (10 mg/kg) in the 2-hour infusion study, but ponezumab was not detected in the CSF in the 10-minute infusion study [67–69]. No antidrug antibodies were detected in either study [69]. In the 2-hour infusion study, there were dose-dependent increases in CSF beta amyloid_1−*x *_ and increases from baseline to day 29 in CSF beta amyloid_1−*x *_ and CSF beta-amyloid_1−42_ with the 10 mg/kg dose [68, 69]. Mass spectrometry following immunoprecipitation identified elevated levels of beta-amyloid_1−40_ and beta-amyloid_11−40_ in CSF following a single dose of 10 mg/kg ponezumab in patients with mild-to-moderate AD [71]. In Japanese patients with mild-to-moderate AD, single-dose ponezumab (0.1–10 mg/kg) showed similar safety and pharmacokinetic profiles as in Western patients [72, 73]. Studies of multiple-dose ponezumab in patients with mild-to-moderate AD are currently in progress (NCT00722046; NCT01125631), and a phase 1 study investigating the effects on single-dose ponezumab on beta amyloid in AD patients and in healthy volunteers is currently recruiting patients (NCT01005862).

#### 5.2.3. Gantenerumab

Gantenerumab (RO4909832/R1450/RG1450), another monoclonal antibody that targets beta amyloid, is currently in clinical development. A phase 1, multiple ascending dose study of gantenerumab in patients with AD has been completed (NCT00531804), while a phase 2 study in patients with prodromal AD is currently recruiting patients (NCT01224106).

#### 5.2.4. BAN2401

BAN2401 is a humanized monoclonal antibody that targets beta-amyloid protofibrils [74]. A phase 1 single- and multiple-ascending dose study of BAN2401 in patients with mild-to-moderate AD is currently recruiting patients (NCT01230853).

#### 5.2.5. Intravenous Immunoglobulin

Putative clinical efficacy data for intravenous immunoglobulin (IVIG) have been reported in 2 small open-label studies. Dodel et al. administered monthly IVIG to 5 AD patients over a 6-month period and demonstrated a decrease in total CSF beta-amyloid levels and an increase in total beta-amyloid serum levels, with no change in beta amyloid_1−42_ in either compartment [75]. The authors reported a slight improvement in mean ADAS-cog and MMSE scores [75]. The second study involved the administration of IVIG to 8 patients with mild AD and demonstrated a dose-proportional increase in serum anti-beta-amyloid antibodies and a decrease in CSF beta amyloid [76]. The CSF beta-amyloid changes were transient, reverting to baseline levels after the discontinuation of IVIG infusions and decreasing again when infusions were restarted [76]. Mean MMSE scores increased during the first 6 months of IVIG, declined when infusions were withheld, and stabilized when infusions were restarted [76]. There was no placebo group in either of these 2 studies, sample sizes were very small, and no brain MRI scans were performed [75, 76]. No serious treatment-emergent AEs were reported [75, 76]. Another study employed a retrospective case-control analysis to demonstrate that previous treatment with IVIG was associated with a reduced risk of AD development [77]. The possible benefits of IVIG, if any, would likely be attributed to the presence of naturally occurring anti-beta-amyloid antibodies in human plasma [26]. Two IVIG clinical trials have been completed (NCT00299988; NCT00812565), and 2 are currently recruiting patients: one phase 2 study (NCT01300728) and one phase 3 trial (NCT00818662).

## 6. Conclusions and Future Directions

More than 100 years after the initial description of AD and the identification of beta amyloid as a key pathologic component, the search for effective anti-beta-amyloid therapies continues, and immunotherapeutic approaches are poised at the front lines of the anti-beta-amyloid battle. Although the preclinical literature is resplendent with examples of effective beta-amyloid clearance, initial attempts to translate these early successes into safe and effective AD therapies were marred by the development of serious and severe side effects in some patients. The next generation of immunotherapies, both active and passive, must demonstrate an acceptable safety profile and the ability to clear beta amyloid, ultimately slowing or halting clinical disease progression. As several pivotal clinical trials in patients with mild-to-moderate AD near completion, studies in patients with mild cognitive impairment/prodromal AD are just beginning, with the hope that targeting beta amyloid earlier in the disease process will provide better clinical outcomes.

##  Conflicts of Interest

Editorial/medical writing support was provided by Benjamin R. Houghtaling, PhD, at Phase Five Communications Inc. and was funded by Pfizer Inc. and Janssen Alzheimer Immunotherapy Research & Development, LLC. KL and RB are employees of Pfizer Inc. JMR was an employee of Pfizer Inc. at the time this study was conducted. EL is an employee of Janssen Alzheimer Immunotherapy Research & Development, LLC.

## Figures and Tables

**Table 1:** Active immunotherapy agents.

Drug name	Sponsor(s)	Mechanism of action	Status	Key clinical data	Source of key clinical data
ACC-001 (vanutide cridificar)	JANSSEN Alzheimer Immunotherapy Research & Development, LLC.; Pfizer Inc.	Multiple copies of A*β*_1-7_ peptide linked to a nontoxic variant of diphtheria toxin	Phase 2	No clinical data have been presented to date	—
AD02	AFFiRiS AG	Short (6 aa) peptide mimicking parts of the native A*β* N-terminus sequence	Phase 2	Phase 1 safety data support proof-of-concept for improved safety profile using AFFITOPE technology	[48]
CAD106	Novartis, Inc.	A*β*_1-6_ peptide coupled with Q*β* carrier	Phase 2	In a phase 2a study, CAD106 showed a favorable safety profile and antibody response in 20/22 patients with mild AD	[45]
V950	Merck & Co.	Multivalent A*β* vaccine	Phase 1	No clinical data have been presented to date	—

A*β*: beta amyloid; AD: Alzheimer's disease.

**Table 2:** Passive immunotherapy agents.

Drug name	Sponsor(s)	Mechanism of action	Status	Key clinical data	Source of key clinical data
Bapineuzumab	JANSSEN Alzheimer Immunotherapy Research & Development, LLC.; Pfizer Inc.	Humanized mAb that targets the N-terminal region of A*β*	Phase 3	No significant differences compared with placebo in primary outcomes (ADAS-Cog or DAD); potential treatment differences based on ApoE4 carrier status	[51]
IVIG (Gammagard)	Baxter International Inc.	Intravenous Ig; contains antibodies against A*β*	Phase 3	Significant differences compared with placebo in primary outcome measures (ADAS-Cog and ADCS-CGIC)	[78]
Solanezumab	Eli Lilly and Company	Humanized monoclonal antibody against the mid-domain of A*β*	Phase 3	Favorable safety profile: no evidence of meningoencephalitis, microhemorrhage, or ARIA-E	[60]
Gantenerumab	Hoffmann-La Roche, Inc.	Monoclonal antibody that targets A*β*	Phase 2	No clinical data have been presented to date	—
IVIG (Octagam)	Octapharma AG	Intravenous Ig; contains antibodies against A*β*	Phase 2	No clinical data have been presented to date	—
IVIG (Newgam)	Sutter Health	Intravenous Ig; contains antibodies against A*β*	Phase 2	No clinical data have been presented to date	—
Ponezumab	Pfizer Inc.	Humanized IgG2deltaA monoclonal antibody that binds to amino acids 33–40 of the A*β*_1−40_ peptide	Phase 2	2 phase 1 studies showed favorable safety profiles, with no microhemorrhage, ARIA-E, or encephalitis	[67, 68]
BAN2401	Eisai Co., Ltd.	Humanized monoclonal antibody that selectively recognizes and eliminates A*β* protofibrils	Phase I	No clinical data have been presented to date	—

A*β*: beta amyloid; ApoE4: apolipoprotein E4A; DAS-Cog: Alzheimer's Disease Assessment Scale-Cognitive Subscale; DAD: Disability Assessment for Dementia; Ig: immunoglobulin.
